# A Video-Based Communication Intervention for Fecal Ostomy Surgery (CI-oSurg): Protocol for Open Pilot Testing to Improve Intervention Acceptability and Feasibility

**DOI:** 10.2196/60575

**Published:** 2024-11-15

**Authors:** Christy Elaine Cauley, Atziri Rubio, Mary Brindle, Zara Cooper, Ana-Maria Vranceanu, Christine S Ritchie

**Affiliations:** 1 Department of Surgery Massachusetts General Hospital Harvard Medical School Boston, MA United States; 2 Mongan Institute Center for Aging and Serious Illness Massachusetts General Hospital Boston, MA United States; 3 Ariadne Labs Harvard School of Public Health Boston, MA United States; 4 Department of Surgery Brigham and Women's Hospital Harvard Medical School Boston, MA United States; 5 The Center for Surgery and Public Health Brigham and Women's Hospital Boston, MA United States; 6 Marcus Institute for Aging Research Boston, MA United States; 7 Department of Psychiatry Massachusetts General Hospital Harvard Medical School Boston, MA United States; 8 Mongan Institute Center for Aging and Serious Illness Massachsuetts General Hospital Boston, MA United States

**Keywords:** fecal ostomy, distress, open pilot, fecal ostomy surgeryl CI-oSurg, intervention acceptability, biopsychosocial outcomes, psychosocial support, ostomy care

## Abstract

**Background:**

Approximately 100,000 patients undergo fecal ostomy operations annually across the United States. This patient population experiences high surgical complication rates and poor biopsychosocial outcomes. Surgical teams are not trained to address the psychosocial needs that often arise during recovery after fecal ostomy surgery.

**Objective:**

This study aims to refine and establish the acceptability and usability of the Communication Intervention for fecal ostomy Surgery (CI-oSurg), a web-based communication intervention aimed at reducing distress among patients recovering from ostomy surgery.

**Methods:**

We describe the proposed study design, methodology, and training protocol. We will conduct an open pilot (n=24 patients and n=8 clinicians) of video-based training to first identify the level and types of distress patients are experiencing. Next, patients will view web videos that address frequent challenges faced by ostomy patients, considering practical management and emotional and adaptation concerns. Qualitative one-to-one semistructured interviews will be conducted with participants to explore the acceptability and feasibility of the program and refine the intervention and study procedures.

**Results:**

This study has been approved by the Mass General Brigham Institutional Review Board. Study funding has been obtained, and recruitment is planned for the fall of 2024.

**Conclusions:**

Through this study, we will refine CI-oSurg, a web-based communication intervention focused on reducing distress after ostomy surgery, to improve intervention acceptability and usability. These improvements will allow us to establish the usability and acceptability of the intervention before efficacy testing to determine the ability of this intervention to reduce distress after fecal ostomy surgery.

**Trial Registration:**

ClinicalTrials.gov NCT06320002; https://clinicaltrials.gov/study/NCT06320002

**International Registered Report Identifier (IRRID):**

PRR1-10.2196/60575

## Introduction

Approximately 100,000 patients undergo fecal ostomy operations annually across the United States [1]. This patient population experiences high surgical complication rates (up to 37%), including surgical site infections, dehydration, and hospital readmissions [2]. Patients undergoing fecal ostomy surgery have poor biopsychosocial outcomes, including decreased quality of life and caregiver burden [3-7]. Surgical teams are not trained to address the psychosocial needs that often arise during recovery after fecal ostomy surgery.

Current colorectal surgery guidelines support the use of interventions to improve biopsychosocial outcomes after ostomy surgery based on expert opinion [8]. Despite these recommendations, a study by Follick et al [9] highlights the lack of adequate ostomy support, with 46% of ostomates reporting that they needed more information than was provided in the time after surgery, with 40% indicating that their spouse needed additional information as well. Furthermore, 45% indicated that more information would have helped them deal more effectively with the emotional effects of their ostomy. Despite this need for more and better information, enhanced recovery after surgery interventions, and the focus of hospital systems on increased throughput, the time spent in the hospital setting is decreased. This limits the time for education of patients and care partners. There is a lack of evidence-based interventions to address distress in the postoperative period for fecal ostomy patients.

The overall goal of our work is to improve biopsychosocial outcomes of patients recovering from fecal ostomy surgery. This study protocol is part of a larger research program to develop, refine, and establish the efficacy of Communication Intervention for fecal ostomy Surgery (CI-oSurg), a video-based communication intervention to support patients and surgical teams during recovery from fecal ostomy surgery. This study aims to elicit and systematically evaluate key stakeholder perspectives on the acceptability and usability of a video-based communication intervention for fecal ostomy surgery, including content and procedures (eg, recruitment and screening methods, eligibility, timing of the intervention, and intervention delivery). Our hypothesis is that iterative improvements to the intervention (ie, CI-oSurg) will improve the acceptability and usability of the intervention before efficacy testing.

## Methods

### Study Design

**Figure 1:**
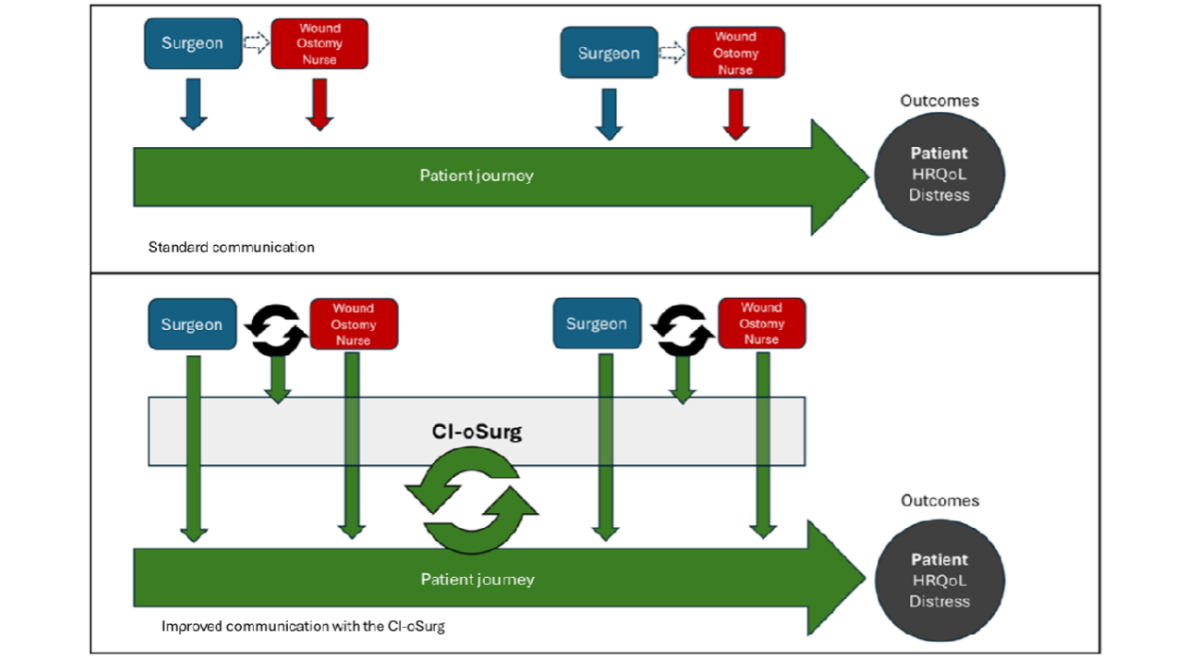
Concept model for improving communication after surgery. CI-oSurg: Communication Intervention for fecal ostomy Surgery; HRQoL: health-related quality of life.

In this study, we will perform open pilot testing of the CI-oSurg intervention with 8 surgical team clinicians (ie, surgeons who perform fecal ostomy operations, inpatient surgical nurses, outpatient surgical advanced practice practitioners, and ostomy nurses) and 24 fecal ostomy patients at a single academic surgical clinic until acceptability is improved [10,11]. Clinicians and patients will use the communication intervention to augment routine surgical care communication, see Figure 1.

**Figure 2:**
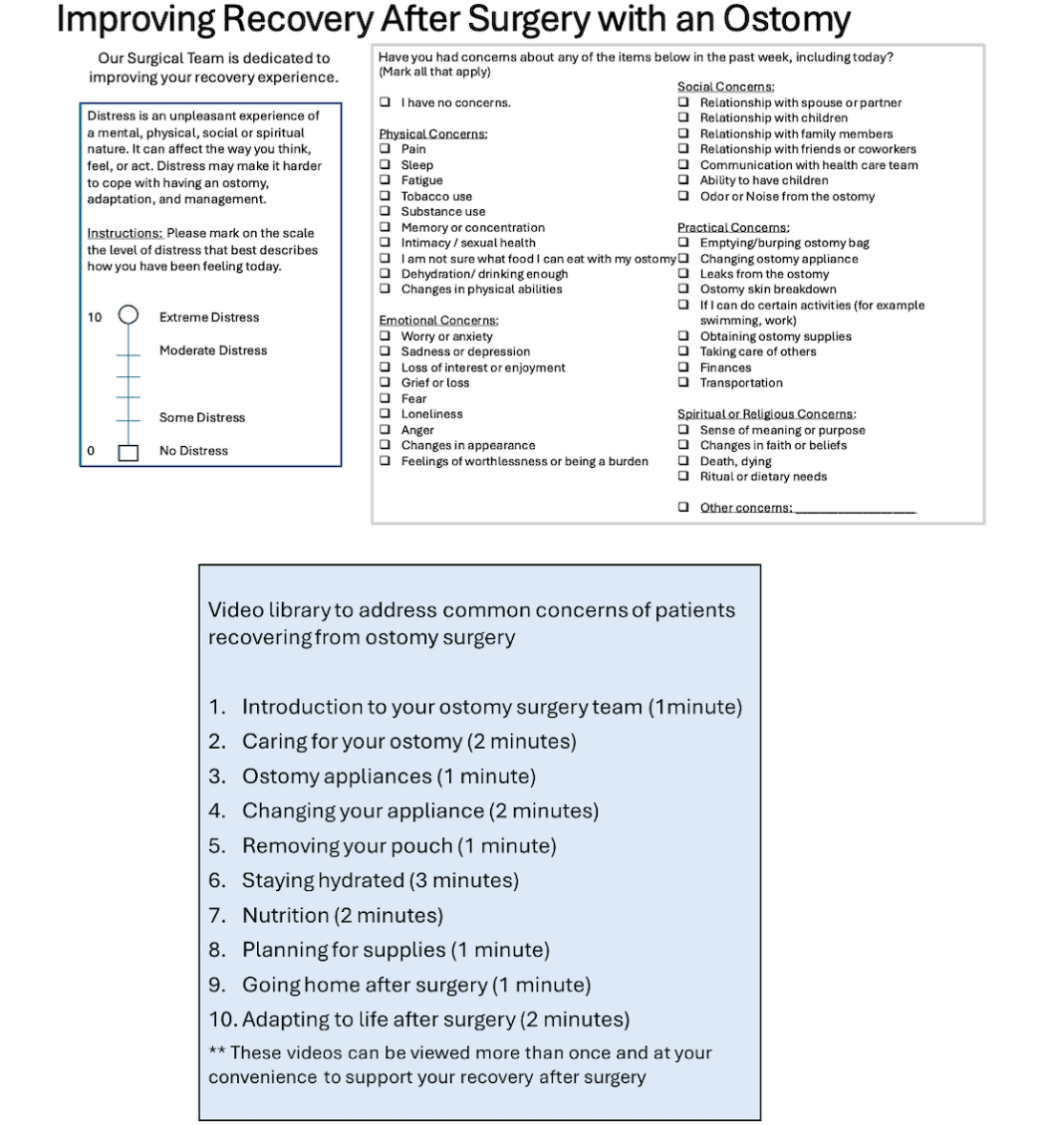
Overview for patients of the Communication Intervention for fecal ostomy Surgery (CI-oSurg) prototype.

The intervention prototype was developed based on qualitative interviews with patients recovering from elective ostomy surgery and focus groups of clinicians experienced in the care of this surgical patient population. The intervention includes a modified version of the National Comprehensive Cancer Network Distress Management Tool [12] and video support materials. The modified distress tool includes a description of distress: an unpleasant experience of a mental, physical, social, or spiritual nature that can affect the way one thinks, feels, or acts. Next, a single distress thermometer question is asked to report the patient’s level of distress on a scale of 0 (no distress) to 10 (extreme distress). Finally, patients complete a problem list to identify concerns they experienced over the past week across domains of physical, emotional, social, practical, and spiritual or religious concerns. After completing this modified distress tool, patients are provided a list of support videos that address the most frequent areas of concern of patients recovering from ostomy surgery (more details in Figure 2). After exposure to the intervention, participants will complete cognitive interviews to provide feedback on the intervention content and protocol to improve acceptability and usability in the surgical setting, as well as a brief survey within 30 days of their operation.

### Recruitment, Screening, and Setting

We will recruit adult patients (aged 18 years or older) scheduled for elective surgery with a fecal ostomy from the outpatient surgical clinic at a single tertiary academic medical center. We will perform purposeful sampling to ensure that half of the patients are aged 65 years or older to understand the acceptability and usability of the intervention across the aging continuum. The potential participants will be identified by the surgical team and research assistant through medical chart review. The research assistant will then complete screening and consent procedures before enrollment. Eligible participants will be (1) fluent in English, (2) willing and able to participate in a live interview or survey, and (3) undergoing elective fecal ostomy surgery. Exclusion criteria include severe cognitive impairment (eg, advanced dementia, stroke, serious mental illness, or altered mental status due to septic shock), causing an inability to perform teach-back for consent, and ensuring meaningful participation in study interviews [13].

We will recruit 8 adult clinicians who participate in the care of patients with a fecal ostomy. Potential participants will be recruited from the same single tertiary academic medical center through surgical team meetings and email. Potential participants will include surgeons who perform fecal ostomy operations, inpatient surgical nurses, outpatient surgical advanced practice practitioners, and ostomy nurses. The research assistant will complete screening and consent procedures before enrollment. Clinician participants will review the CI-oSurg intervention and training module, a 30-minute in-person didactic session with role-play to ensure an understanding of the intervention and study purpose.

The intervention will be provided on general and gastrointestinal surgical inpatient floors at a Boston tertiary care hospital where a high volume of colorectal surgery is performed. These surgical inpatient floors are staffed by nurses experienced in caring for patients with fecal ostomies who have access to onsite ostomy nursing staff during weekdays.

### Study Intervention

Participating clinicians will be trained in the CI-oSurg intervention and protocol using the CI-oSurg training module. The training module will consist of a 30-minute in-person didactic session with role-play of intervention components. This will occur in the inpatient surgical floor conference room to provide participants with a convenient and familiar setting where they will execute the intervention. We will provide flexible scheduling and reschedule sessions as needed to increase clinician recruitment. During this module, participants will learn the potential benefits of psychological support for patients recovering from ostomy surgery and address the distress patients often experience during the recovery process. Participants will view the intervention, including intervention video content.

In total, 24 consenting patient participants will complete the CI-oSurg intervention assessment and management video series 1-3 days after fecal ostomy surgery while admitted to the hospital. The intervention content and protocol will be refined iteratively with each group of 4 to 6 participants based on qualitative feedback from the interviews. For example, participants will be asked if the content addresses concerns using understandable terms and images and if they would like to repeat the intervention in the hospital or at home through a web-based platform on a personal tablet or device. Patients might request changes to the images of the video content, the size of the font, or the speed of audio recordings.

Using a semistructured interview guide, participants will then undergo an approximately 30-minute in-person cognitive interview before discharge from the hospital and within 2 weeks of surgery. Interview guide content is designed to gather feedback on the proposed CI-oSurg content and protocol. Specifically, this study is designed to gather participants’ perspectives on the following: (1) acceptability of the CI-oSurg content components, (2) acceptability of clinician training sessions, (3) useability of the intervention with the surgical team and existing clinical staff, and (4) proposed recruitment procedures for a future clinical trial.

### Study Procedures

To improve the credibility of results, interviews will be performed by trained qualitative researchers, audio recorded, and transcribed. Rapid data analysis will be performed to allow changes to be quickly made to the intervention to improve acceptability and feasibility in the busy inpatient surgical setting before in-depth coding and analysis. Final study data will undergo in-depth coding and analysis to ensure the credibility of findings, with double coding of at least 20% of interviews to ensure consistency. A hybrid inductive-deductive approach [14] will be used for qualitative coding, and a modified framework method for thematic synthesis in the final analysis [15-17]. Our team will use an iterative refinement of the CI-oSurg intervention and protocol based on ongoing data collection and rapid data analysis, consistent with previous studies [18].

### Data Collection

Data from all participants will be collected using REDCap (Research Electronic Data Capture; Vanderbilt University) tools hosted at Massachusetts General Hospital (MGH) and a HIPAA (Health Insurance Portability and Accountability Act)-approved electronic data capture system [19]. Demographic data will be obtained upon study enrollment (ie, age, sex, race and ethnicity, education, and occupation). In addition, the World Health Organization Brief Quality of Life Evaluation [20] and Hospital Anxiety and Depression Scale [21] data will be collected from patient participants 1 month after the surgery date to evaluate retention for future clinical trial planning. Assessment responses from the intervention will be collected by the research assistant as part of the CI-oSurg intervention. The Client Satisfaction Questionnaire [22] will be administered within 1 month of intervention use. In addition, we will conduct qualitative interviews with patients and clinicians. Interviews will be 1:1 and will last approximately 30 minutes to allow adequate time for discussion. The interview will follow a semistructured interview guide with open-ended prompts and questions surrounding domains including (1) perceptions of fecal ostomy care after surgery, (2) feedback on the CI-oSurg intervention procedures and content, and (3) ways of maximizing acceptability and feasibility of use. The interviewer will follow the guide with further probes to develop a comprehensive understanding of the participants’ perspectives (more details in Multimedia Appendix 1).

Interviews will be audio-recorded and performed by a trained research assistant with expertise in qualitative research methods. Recordings will be transcribed and reviewed with rapid data analysis using a template developed by the research team. The template will be used to inform formal coding and intervention refinement with observations within key interview domains, critical participant statements, and other important observations. The research team will work to generate information on clinical team perspectives regarding the acceptability and usability of the intervention procedures and content.

### Data Analysis

Audio recordings will be transcribed and deidentified. Evaluation of deidentified data using rapid analysis will be performed in Microsoft Excel to allow rapid updates to the intervention based on participant feedback. A hybrid deductive-inductive approach to qualitative data analysis will be used, guided by a modified framework method [14-17]. Our deductive approach will be based on our interview guide, rapid analysis template (more details in Multimedia Appendix 2), and codebook domains. These are influenced by our previous research on patient perspectives on challenges faced by patients undergoing fecal ostomy surgery and the findings from our clinician focus groups for prototype development. For example, we will include prompts in the interview guide that assess the perspectives of patients and clinicians in evaluating social support and adapting to life after surgery.

Transcripts will undergo rapid data analysis with summarizing data matrices to determine real-time data saturation before conducting more extensive qualitative analyses [23]. Rapid data analysis bypasses the process of in-depth coding and organizes data immediately following qualitative interviews based on interview script templates [24]. Rapid analysis will be used to enact changes to the CI-oSurg intervention and protocol until thematic saturation is reached, with 32 participants anticipated based on past studies and practical considerations [25]. Discrepancies in coding or analysis will be resolved through discussions with the larger research team to ensure consistency and accuracy.

### Ethical Considerations

This study was approved by the Mass General Brigham Hospital Institutional Review Board (2023P003564). Informed consent will be obtained verbally during enrollment and during qualitative interviews. Participant data will be deidentified and stored securely on HIPAA-compliant servers. Participants will not receive compensation for participation.

## Results

The study has been approved by the Mass General Brigham Institutional Review Board and registered with ClinicalTrials.gov. Study funding was obtained in the summer of 2022. Open pilot recruitment is expected to begin in fall 2024. Completion is anticipated by winter 2024, with plans for dissemination of study findings at national surgical meetings in the spring of 2025, with peer-reviewed publications to follow.

## Discussion

Findings from this study will improve the acceptability and usability of a web-based communication intervention for fecal ostomy surgery to address challenges leading to distress during fecal ostomy surgery recovery. Recovery after fecal ostomy surgery requires practical management changes (ie, changing an ostomy appliance and monitoring output) as well as emotional adaptation to life with an ostomy. This novel intervention leverages existing technology and resources in surgical clinics to provide scalable, psychologically informed care aimed at improving the biopsychosocial outcomes of patients recovering from fecal ostomy surgery.

Current colorectal surgery guidelines support the use of interventions to improve biopsychosocial outcomes after ostomy surgery based on expert opinion [26]. Communication interventions have been used in some surgical practices (ie, oncology) to identify distress [27,28]. However, these interventions are not currently used in benign surgical settings, and management strategies to address ostomy patient needs are lacking. The intervention refined in this study will evaluate the level of distress, identify the potential needs of patients recovering from ostomy surgery, and provide management steps. By addressing common concerns identified by patients and clinicians interviewed by our team during prototype development, we hypothesize that the intervention will reduce postoperative patient distress during future efficacy testing.

The intervention under development in this study, CI-oSurg, is novel because current surgical care models limit the integration of psychologically informed care. While psychiatrists are involved in the care of select surgical patient populations (preoperative bariatric surgery screening), there is a lack of psychological support for patients facing and recovering from fecal ostomy surgery. Providing individual counseling to all fecal ostomy patients is unlikely to be feasible due to resource constraints [29]. However, screening for distress, anxiety, and depression before and after ostomy surgery is a key first step in identifying the needs of this high-risk surgical population [9,30,31]. The use of a distress screen in this communication intervention with a problem list to compartmentalize challenges will provide surgical teams with a better understanding of specific, individualized interventions needed to support patients during recovery after ostomy surgery.

In this study, we will ensure the recruitment of older adults facing fecal ostomy surgery to understand the unique needs of this growing patient population. Older adults undergoing fecal ostomy surgery have poor biopsychosocial outcomes, including a high rate of permanent ostomy formation, poor quality of life, and high levels of caregiver burden [3-7,31-34]. A study by Bosshardt [35] reports high rates of mortality and permanent ostomy among patients aged 70 years or older. Among aging adults, poor outcomes can be linked to geriatric conditions. For example, arthritis or sensory impairments can impact patients’ ability to perform practical management tasks required for independent ostomy care, and polypharmacy and neurocognitive impairment contribute to dehydration. If older adults note new challenges, such as medication management for dehydration, we will refine the intervention to address these needs before finalizing prototype content. This focus on older adult ostomy patients will provide key insights to improve the care of this growing surgical population [36].

During this open pilot phase, certain barriers may arise to using this intervention in the busy inpatient surgical setting. This study will identify and address barriers to intervention acceptability, such as clinicians’ limited time or unfamiliarity with distress screening strategies before efficacy testing [37,38]. In addition, the usability of certain intervention components might need to be modified (ie, using hospital-provided tablets vs personal devices). Using a human-centered design approach to obtain information on these potential barriers is a critical step in intervention development to optimize the intervention acceptability before efficacy testing.

This study should be considered in light of important limitations. The web-based intervention content is only in English; therefore, lack of English fluency is an exclusion criterion. Future work will need to be performed to develop and evaluate the acceptability and usability of a culturally adapted intervention in diverse populations considering different languages and ethnic groups. Next, the study will be performed at a single institution in the northeastern United States. Clinicians practicing in different hospital settings might find unique challenges when using interventions with different clinical demands or patient populations.

### Conclusion

Through this open pilot study, we will refine CI-oSurg, a web-based communication intervention focused on addressing the biopsychosocial needs of patients recovering from ostomy surgery. Current colorectal surgery guidelines support the use of interventions to improve outcomes after ostomy surgery; however, there is a paucity of evidence-based interventions to address the needs of this population. This study is the next step in establishing the usability and acceptability of the CI-oSurg intervention. Future efficacy testing will provide surgical teams with an evidence-based, scalable communication intervention that addresses the biopsychosocial needs of this large surgical patient population.

## Appendix

Multimedia Appendix 1 Semistructured interview guides.

Multimedia Appendix 2 Rapid analysis template.

Multimedia Appendix 3 Peer-review report by the Career Development for Clinicians/Health Professionals Study Section - National Institute on Aging Initial Review Group - Career Development for Clinicians and Health Professional Awards (National Institutes of Health, USA).

## Abbreviations

CI-oSurg: Communication Intervention for fecal ostomy Surgery
HIPAA: Health Insurance Portability and Accountability Act
MGH: Massachusetts General Hospital
REDCap: Research Electronic Data Capture

## Data Availability

The data sets generated during and/or analyzed during this study will be available from the corresponding author on reasonable request.
