# Supplementary material for: A Video-Based Communication Intervention for Fecal Ostomy Surgery (CI-oSurg): Protocol for Open Pilot Testing to Improve Intervention Acceptability and Feasibility
Source: JMIR Res Protoc. 2024 Nov 15;13:e60575. doi: 10.2196/60575 (PMC11607551; doi:10.2196/60575)
Supplement: Multimedia Appendix 1 [file resprot_v13i1e60575_app1.docx]

Multimedia Appendix 1. Semistructured interview guides.

Patient guide-As part of your hospital stay you were asked to participate in an intervention to improve ostomy patient care, which included a survey about problems that caused distress during your recovery and videos to help you better manage your ostomy and adapt to life at home after surgery (show the distress one-page survey).

1. Recruitment procedures:
   1. What do you think are motivators for patients like you to participate in this type of intervention?
   2. What was your general impression of being recruited for this study?
   3. In your opinion, are there any ostomy patients that would NOT be suitable for this intervention?
2. Intervention acceptability:
   1. Consider the one-page survey with problem list (share screen).
      1. What did you think of this part of the intervention overall?
      2. What did you find helpful with this part of the intervention?
      3. Were there things that you did not like about this part of the intervention?

Were there things that you wish were included in this part of the intervention (for example different problems for the list)?

- 1. Consider the videos (show list of videos)
     1. What did you think of this part of the intervention overall?
     2. What did you find helpful with this part of the intervention?
     3. Were there things that you did not like about this part of the intervention?
     4. Were there things that you wish were included in this part of the intervention (for example different videos)?

1. Intervention feasibility
   1. Did you have any difficulty accessing the survey or viewing the videos?
   2. Did you use the videos after you were discharged from the hospital?
      1. What did you think about the length of time for the videos?
      2. What did you think about the number of videos and surveys?
   3. What did you think about the timing of this interview and completing the quality of life survey?
   4. Were there barriers to completing any portion of the study?
2. Intervention useability
   1. Would you prefer to use a hospital provided tablet to view the videos or a personal device?
   2. Would you prefer having the videos available to view at your convenience or to have a staff member present to help you open the videos
   3. How would sharing the video content or survey findings with a care-partner or family member affect your recovery?
3. Retention procedures:
   1. Did you have any difficulty accessing the survey to complete at the end of the study?
   2. How can we improve completion of surveys or interviews with patients several weeks after surgery for future studies?
4. Wrap up

Is there anything else that we did not ask that you would like to share?

Thank you for participating. [*Instruct to press the red hang-up button.*]

Clinician guide: As part of patients’ hospital stay they will be asked to participate in an intervention to improve ostomy patient care, which includes a survey about problems that may cause distress during surgical recovery and videos to help better manage their ostomy and adapt to life at home after surgery (show the distress one-page survey).

1. Recruitment procedures:
   1. What do you think are motivators for patients like the ones you care for to participate in this type of intervention?
   2. What was your general impression of patients being recruited for this study?
   3. In your opinion, are there any ostomy patients that would NOT be suitable for this intervention?
2. Intervention acceptability:
   1. Consider the one-page survey with problem list (share screen).
      1. What did you think of this part of the intervention overall?
      2. What did you find helpful with this part of the intervention?
      3. Were there things that you did not like about this part of the intervention?
      4. Were there things that you wish were included in this part of the intervention (for example different problems for the list)?
   2. Consider the videos (show list of videos)
      1. What did you think of this part of the intervention overall?
      2. What did you find helpful with this part of the intervention?
      3. Were there things that you did not like about this part of the intervention?
      4. Were there things that you wish were included in this part of the intervention (for example different videos)?
3. Intervention feasibility
   1. Do you think patients will have difficulty viewing the videos?
      1. What do you think about the length of time for the videos?
      2. What do you think about the number of videos and surveys?
   2. What do you think about the timing of this interview and completing the quality of life survey?
   3. Were there barriers to completing any portion of the study?
4. Intervention useability
   1. Would it be best to use a hospital provided tablet to view the videos or a personal device for inpatients?
   2. Do you think patients would prefer having the videos available to view at their convenience or to have a research member/staff present to help patients open the videos
   3. How would sharing the video content or survey findings with a care-partner or family member affect patient recovery?
5. Retention procedures:
   1. Did you have any difficulty accessing the survey to complete for this study?
   2. How can we improve completion of surveys or interviews with patients several weeks after surgery for future studies?
6. Wrap up

Is there anything else that we did not ask that you would like to share?

Thank you for participating. [*Instruct to press the red hang-up button.*]
