# Supplementary material for: A Video-Based Communication Intervention for Fecal Ostomy Surgery (CI-oSurg): Protocol for Open Pilot Testing to Improve Intervention Acceptability and Feasibility
Source: JMIR Res Protoc. 2024 Nov 15;13:e60575. doi: 10.2196/60575 (PMC11607551; doi:10.2196/60575)
Supplement: Multimedia Appendix 2 [file resprot_v13i1e60575_app2.docx]

| **Date:**  **Prepared by:**  **Interviewer:** |
| --- |
| **Stakeholder Information:**  ***Occupation:***  ***Years of Experience Working with surgery patients (if clinician):***  ***Other relevant characteristics****:* |
| **Domain 1: Usual Surgical Care after fecal ostomy surgery** |
| **CI-oSurg Intervention Recommendations and Procedures**  **General Impressions** |
| **Video Content** |
| **CI-oSurg Procedures**  **Screening Procedures**  **Recruitment Procedures**  **Maximizing Feasibility and Acceptability Outcomes** |
| **Important observations and reflections**  ***Broad themes and topics of interest in the interview*** *(e.g., things not covered in rapid data analysis domains*)**:**  **Important quotations** |
| **Interviewee analytic notes + reflexivity** (e.g., any aspects of researchers’ identity, beliefs, social positioning and how they might influence the interview content and observations—consider how your field notes are your own *interpretations* of the interactions that took place)**:** |
| **Behind-the-scenes information (nonverbal information; etc.):** |

Multimedia Appendix 2. Rapid analysis template.
