# Supplementary material for: A Video-Based Communication Intervention for Fecal Ostomy Surgery (CI-oSurg): Protocol for Open Pilot Testing to Improve Intervention Acceptability and Feasibility
Source: JMIR Res Protoc. 2024 Nov 15;13:e60575. doi: 10.2196/60575 (PMC11607551; doi:10.2196/60575)
Supplement: Multimedia Appendix 3 [file resprot_v13i1e60575_app3.pdf]

HARVARD CATALYST
Harvard Clinical & Translational Science Center

HARVARD UNIVERSITY

August 29, 2022

Christie Elaine Cauley, MD, MPH
Massachusetts General Hospital
Department of Surgery
15 Parkman Street
WACC 460
Boston, MA 02114

Via E-Mail

Dear Dr. Cauley:

On behalf of Harvard Catalyst, we are pleased to inform you that you have been selected for a KL2/Catalyst Medical Research Investigator Training (CMeRIT) award. Congratulations on this achievement.

Your award is being made under the CMeRIT portion of the program and is a non-federal two-year award, requiring that you commit 50% of your [redacted] which includes your research as well as [redacted] This award will provide [redacted] (note that salary is capped at [redacted]

We expect to assist you in developing the best educational program to match your career goals. It is our hope that this award will provide you with the necessary support and guidance as you continue to develop into an independent clinical and translational scientist.

Please respond by September 6, 2022 to confirm your acceptance of this award. Your award will commence on **October 1, 2022**.

Please direct your response to:

[redacted]

Please see below for additional terms of this award.

Again, congratulations and we look forward to working with you.

Sincerely,

[redacted]

HARVARD CATALYST
Harvard Clinical & Translational Science Center

HARVARD UNIVERSITY

Please review the terms and requirements of your award:

1. [redacted]
2. Assure tha[redacted] is allocated to the award.
3. Attend monthly KL2/CMeRIT Lab/Faculty Meetings, as scheduled.
4. Attend Harvard Catalyst Research Day in Spring 2023 – official date forthcoming.
5. Present your research project at a KL2/CMeRIT Lab/Faculty Meeting on a date to be determined.
6. Participate in a 60-minute-long Mentorship Advisory Committee meeting in which you will receive feedback on your research project.
7. Complete evaluations, as requested, for the Harvard Catalyst program evaluation purposes. **Outreach for data collection will continue in the years after your appointment has ended.**
8. A mandatory component of the program is participation in the Harvard Catalyst Grant Review and Support Program (GRASP). GRASP is a multi-year program that guides KL2/CMERIT and K awardees to understand the grant writing process, gain new skills, and write competitive grant applications to ultimately achieve research independence. Participation in GRASP improves the chances of obtaining independent research funding with the use of project management tools and long-term grant-writing guidance. The GRASP orientation workshop will be held VIRTUALLY via Zoom this fall. The workshop will be broken down into three, 2-to-3-hour remote learning sessions.

   Dates and times are as follows:

   Wednesday, September 14, 2022 from 9:00am – 12:00pm EST
   Wednesday, September 21, 2022 from 9:00am – 12:00pm EST
   Wednesday, September 28, 2022 from 9:00am – 12:00pm EST

   Following the fall sessions, there will be a required four-part grant writing module in the winter of 2023. The module will be broken down into four, 2-to-3-hour remote learning sessions via Zoom.

   Tuesday, February 14, 2023 from 9:00am – 11:30am EST
   Tuesday, February 21, 2023 from 9:00am – 11:30am EST
   Tuesday, February 28, 2023 from 9:00am – 11:30am EST
   Tuesday, March 7, 2023 from 9:00am – 11:30am EST

   If you have any questions about GRASP, please contac[redacted]

Thank you for your participation, and congratulations!

[redacted]

SUMMARY STATEMENT
(Privileged Communication)

Release Date: 07/14/2023
Revised Date:

Application Number: 1K23AG084876-01

Principal Investigator

CAULEY, CHRISTY ELAINE

Applicant Organization: MASSACHUSETTS GENERAL HOSPITAL

Review Group: AGCD-3
Career Development for Clinicians/Health Professionals Study Section
Carrer development for clinicians and health professional awards

Meeting Date: 06/12/2023
Council: OCT 2023
Requested Start: 10/01/2023

Opportunity Number: PA-20-206
PCC: 4AGERMS

Project Title: Improving Biopsychosocial Outcomes of Older Adults Facing Fecal Ostomy Surgery
SRG Action: Impact Score:34
Next Steps: Visit https://grants.nih.gov/grants/next_steps.htm
Human Subjects: 30-Human subjects involved - Certified, no SRG concerns
Animal Subjects: 10-No live vertebrate animals involved for competing appl.
Gender: 1A-Both genders, scientifically acceptable
Minority: 1A-Minorities and non-minorities, scientifically acceptable
Age: 3A-No children included, scientifically acceptable

[redacted]

ADMINISTRATIVE BUDGET NOTE: The budget shown is the requested budget and has not been adjusted to reflect any recommendations made by reviewers. If an award is planned, the costs will be calculated by Institute grants management staff based on the recommendations outlined below in the COMMITTEE BUDGET RECOMMENDATIONS section.

RESUME AND SUMMARY OF DISCUSSION: This application for a Mentored Patient-Oriented Research Career Development Award (Parent K23, Independent Clinical Trial Required) submitted by the Massachusetts General Hospital (MGH), Boston, MA, on behalf of Dr. Christy Cauley, requests five years of support to transition the candidate to an independent clinical research career focusing on improving outcomes in older subjects undergoing gastrointestinal surgery (fecal ostomy). The candidate obtained an MD from Indiana University, an MPH from Harvard University, completed a residency in Surgery at MGH, and a fellowship in Colon and Rectal Surgery at the Cleveland Clinic. Currently an Assistant Professor of Surgery at MGH, Dr. Cauley has published more than 47 peer-reviewed articles, more than 20 in the field of gerontology and surgery, and more than 20 as first author. She is the recipient of professional and grant awards as Principal Investigator (PI), such as, for example a KL2 training fellowship and a Spark Grant. The career development plan (CDP) training goals include clinical geriatrics focused on ostomy care needs, mixed-methods research, and clinical trial design that are well aligned with the research aims. The CDP encompasses a good mix of formal coursework, supporting the selected training areas, scientific meetings, plans for publications, and an appropriate schedule of meetings with the mentors/advisors. The research plan (RP) includes a well conceptualized framework, is well conceived, developed, and includes appropriate analytic plans; it includes three Specific Aims (SA) with the overarching goal to setup, field test, and challenge, in a pilot randomized clinical trial (RCT), a Geriatric Assessment and Management framework for ostomy Surgery (GAM-oSurg) tool to assist in the management of geriatric patients with diverticulitis undergoing surgery to install an ostomy. The multidisciplinary mentorship team is comprised of highly qualified and well-funded advisors who also have excellent track records of mentorship and publications. The team includes the primary mentor, Dr. Ritchie (Professor at MGH; expert in geriatrics and palliative care; highly experienced in mixed-methods research and pragmatic clinical trials; she is the recipient of several mentoring awards); two co-mentors, Drs. Cooper (Chair in Surgery at Brigham and Women's Hospital; trauma surgeon and palliative care physician) and Vranceanu (Associate Professor at MGH; expert in biopsychosocial intervention development, will provide near-peer training); one collaborator, Dr. Eum (Senior Statistical Analyst at Harvard University; expert in statistics). The candidate has also set up a scientific advisory board to help her manage the career development, which includes the primary mentor (Chair), Drs. Russel (UCLA), Brindle (ARIADNE laboratories), and Traeger (MGH). The institutional support stated by Dr. Lillemoe, Chief of the Department of Surgery at MGH, indicates unconditional-on-the-award support for 60%-protected research time, the applicant faculty position and salary, administrative support, and space. The rich environment provided by the Harvard educational and healthcare system is excellent and will properly support the candidate's efforts. Weaknesses of the application include the limited publication record of the candidate with the primary mentor and some redundancy in the coursework choices. The RP shortcomings are moderate and include limited details about many aspects of the proposed research, including the tool under study; lack of a through control group; SA3 that is underdeveloped; lack of involvement of key stakeholders such as dieticians, ostomy nurses, and caregivers; a weak effort toward the recruitment of a diverse pool of participants, and lack of consideration of socioeconomic status (SES) in the designs and analytic plan. The choice of a one-to-one randomization could be unwise; the unit of randomization is not well described; only English-speaking participants will be included in the interviews; and comorbidities and other confound factors are not discussed. The justification for a two-arms' RCT is unclear, blinding is probably not possible, and the role of physical therapy is not considered.

TRAINING IN THE RESPONSIBLE CONDUCT OF RESEARCH: Acceptable. The planned activities satisfy the requirement for training in the responsible conduct of research.

DESCRIPTION (provided by applicant): In this K23 proposal, I outline a comprehensive 5-year training program that will support my transition to independence focused on development and rigorous testing of an intervention to improve integration of geriatric principles into surgical care of older adults undergoing fecal ostomy surgery. This significant and innovative plan is directly tied to my training and career development goals. Background: Fecal ostomy surgery impacts biopsychosocial outcomes of

older adults; however, surgeons are not fully equipped to address geriatric needs that exist among this growing surgical patient population. Developing interventions for surgical teams (i.e., surgeons, nurses, and medical assistants) to address geriatric needs when facing fecal ostomy surgery is an unexplored opportunity to improve biopsychosocial outcomes, including quality of life, independence, and caregiver burden, in this aging patient population. Specific aims and research design: I aim to develop the first version of the Geriatric Assessment and Management framework for ostomy Surgery (GAM-oSurg) intervention and methodology through 1:1 interviews with older adults 30-90 days after fecal ostomy surgery (up to N=30) (Aim 1a), with additional input from focus groups of key clinical stakeholders (surgeons, geriatricians, surgical nurses, and social workers) (N=24) (Aim 1b). Next, I will examine acceptability of GAM-oSurg via an open pilot (N=12 patients, N=4 surgical clinicians) with exit interviews and pre-post assessments. GAM-oSurg intervention and methodology will be refined (Aim 2). Finally, I will examine the feasibility of GAM-oSurg versus enhanced usual surgical care (N=48 patients per study arm) in a pilot RCT following predetermined benchmarks (Aim 3). These findings will inform an efficacy trial through the R01 mechanisms to determine intervention impact on patient quality of life and future studies extending this work to other surgical clinic sites. Training: My aims are supported by training goals to develop expertise in: 1) clinical geriatrics; 2) mixed-methods data integration to inform intervention development and refinement; and 3) clinical trial methodology focusing on efficacy and effectiveness trials. These training goals are support by 1) my expert mentorship team led by my primary mentor Dr. Christine Ritchie (a geriatrician and palliative care physician with expertise in mixed-methods research and clinical trials), 2) a rich institutional environment (Massachusetts General Hospital and Harvard Medical School), and 3) targeted coursework, scientific meetings and planned publications. Relevance to NIA: This K23 is in line with NIA's 2020-2025 Strategic Directions for Research Goal C, to "develop effective interventions to maintain health, well-being, and function and prevent or reduce the burden of age-related diseases, disorders, and disabilities". Impact: As a surgeon with expertise in colorectal diseases I recognize the critical need to improve biopsychosocial outcomes among older adults facing surgery. Experiences gained through this proposal are foundational to my path to research independence as a leader at the intersection of geriatrics and surgery.

PUBLIC HEALTH RELEVANCE: This proposal will lead to the development and preliminary testing of a novel intervention, Geriatric Assessment and Management framework for ostomy Surgery (GAM-oSurg), aimed at addressing unmet needs of older adults facing fecal ostomy surgery. Surgical teams are not fully equipped to address the biopsychosocial needs of the rising number of older adults undergoing fecal ostomy surgery. The proposed intervention includes geriatric assessment and management steps to improve integration of geriatric principles into surgical care of older adults undergoing fecal ostomy surgery and will provide the training necessary to launch my career as an independent surgeon-scientist and leader at the intersection of geriatrics and surgery.

DISCLAIMER: Please note that the following critiques were prepared by the reviewers prior to the Study Section meeting and are provided in an essentially unedited form. Even though there is opportunity for the reviewers to update or revise their written evaluation, based upon the group's discussion, there is no guarantee that individual critiques have been updated after the discussion at the meeting. Therefore, the critiques may not fully reflect the final opinions of the individual reviewers at the close of group discussion or the final majority opinion of the group. Thus, the Resume and Summary of Discussion is the final word on what the reviewers considered critical at the meeting.

CRITIQUE 1:

Candidate: 1
Career Development Plan/Career Goals/Plan to Provide Mentoring: 3
Research Plan: 5

Mentor(s), Co-Mentor(s), Consultant(s), Collaborator(s): 1
Environment and Commitment to the Candidate: 1

Overall Impact:
The overall clinical goal is to develop and rigorously test an intervention to improve integration of geriatric principles into surgical care of older adults undergoing fecal ostomy surgery. This application will lead to the development and preliminary testing of a novel intervention, Geriatric Assessment and Management framework for ostomy Surgery (GAM-oSurg), aimed at addressing unmet needs of older adults facing fecal ostomy surgery. She is a strong candidate who has been well trained, conducted clinical research, and has a strong publication record. The career plans include training in older adults with ostomy, mixed methods research to integrate qualitative and quantitative data, and to acquire knowledge and skills in clinical trials research to evaluate efficacy and effectiveness of interventions. She will achieve these goals with classwork, by caring for patients, and by attending scientific meetings. The research goal is to determine the needs of older adults undergoing fecal ostomy surgery. She will interview key stakeholders to understand patient needs and core interventions. She will be developing and testing GAM-oSurg for usability and acceptability in an open pilot trial and finally feasibility of GAM-oSurg versus Enhanced Usual Surgical Care (EUSC) in a pilot randomized clinical trial (RCT). Several key stakeholders will not be interviewed, limiting the robustness of framework weakens the research goal. The mentors and environment are outstanding.

1. Candidate:
Strengths
- She earned her MD from Indiana University, MPH from Harvard TH Chan SPH. Residency in Surgery MGH, Fellow Colon and Rectal Surgery from the Cleveland Clinic. She is an Assistant Professor of Surgery at MGH since 2019. She is a Core Faculty at the Ariadne Laboratory, Boston, MA.
- She has published over 47 peer-reviewed publications.
- She is the recipient of the KL2/CMERIT Harvard Catalyst and Kornfeld Scholars Program Awards which end in 2024. She completed a Spark Grant as PI, Argosy Foundation (investigator) and APTIMA Health Prism (co-PI) grants.
- She has been funded, well published and shows interest in being a physician-scientist.

Weaknesses
- None noted.

2. Career Development Plan/Career Goals and Objectives:
Strengths
- She will further her training in 1) clinical geriatrics focused on ostomy care needs, 2) mixed methods data integration for intervention development and refinement, and 3) clinical trials to evaluate efficacy and effectiveness.
- She will take courses and intensive training: SA1: Epidemiology of aging; Issues in frailty; SA2: Intro to qualitative research methods for public health; SA3: Principles of clinical trials: medical informatics.
- She will attend the American Geriatrics Society annual scientific meeting every year, the American College of Surgeons Clinical Congress in years 2 and 4 and the American Society of Colon and Rectal Surgeons national conference in years 1, 3, and 5.
- Mentor meetings: primary mentor (weekly), co-mentors (bimonthly), statistics collaborator (monthly), Scientific Advisory Board (annually).
- She will commit 60% effort to K23, 40% to maintaining clinical competency.

Weaknesses
- Possible overlap in course material: 'Introduction to Qualitative and Mixed Methods Research', and 'Qualitative Analysis' versus Introduction to Qualitative Research Methods for Public Health.

3. Research Plan:
Strengths

- SA1: to determine needs of older adults undergoing fecal ostomy surgery through key stakeholder engagement. She will conduct semi-structured interviews of older adults 30-90 days after elective fecal ostomy surgery to evaluate ways to assess and manage perioperative needs. focus groups of clinical stakeholders (surgeons, geriatricians, surgical nurses, and social workers) to identify core intervention components and methodology concerns.
- SA2: to examine usability and acceptability of GAM-oSurg via an open pilot. She will conduct an open pilot (N=12 patients and n=4 surgical clinicians; 16 participants) with exit interviews to address patient and clinician acceptability of the intervention, including attitude toward the intervention and perceived effectiveness.
- SA3: to evaluate feasibility of GAM-oSurg versus Enhanced Usual Surgical Care (EUSC) in a pilot RCT. She will conduct a pilot RCT (n=48 patients per arm and 2 surgical teams per arm) of GAM-oSurg versus EUSC to determine RCT feasibility. Hypothesis: GAM-oSurg will meet a priori benchmarks (e.g., feasibility of recruitment). GAM-oSurg will show feasibility in completion of assessments and initiation of management steps.
- Patients, surgeons, geriatricians, nurses, and social workers will be participating.

Weaknesses

- Caregivers will not be evaluated during this research project. However, the candidate acknowledges their contribution to patient outcomes. They will be evaluated in a subsequent study.
- Evaluation of the patients who decline surgery will occur in future studies. Clearly, they could serve as the control group. In SA3, the enhanced usual care will be the control group.
- Dietitians/nutritionists are not involved. The patient physiologic status is not mentioned. The GI disorder leading to ostomy creation impacts patient's oral intake and weight. The nutritional status of these patients nor co-morbidities are considered.
- Ostomy nurses plays a key role in educating the patient to care for the ostomy are not included.
- GAM was presented as a theoretical construct, something to be developed.

4. Mentor(s), Co-Mentor(s), Consultant(s), Collaborator(s):
Strengths

- Dr. Ritchie, Mentor, Kenneth L Minaker Endowed Chair. Chair and Director Res, Division Palliative Care and Geri Med, Professor Med, (MD, MSPH), mentored over 65 faculty members, 18 pre- and post-doctoral trainees, and 25 career development awardees. She has mentored Dr. Cauley for over 2 years. She has been well funded, well published. Her research focuses on older persons with complex serious illnesses and multimorbidity with an emphasis on persons living with dementia (PLWD) and their caregivers, and on older adults with chronic pain.
  She serves as one of the PI of the National Institute for Nursing Research-funded Palliative Care Research Cooperative (PCRC) and directs the PCRC Investigator Development Center with Drs. Kutner and Pollak. She directs the Mongan Institute Center for Aging and Serious Illness, which is designed to bring together researchers across MGH and the Boston community to improve the well-being of older adults and enhancing the lived experience of those with advanced illnesses.
  She is the recipient of the 2019 University of California San Francisco Academic Senate Distinction in Mentoring Award.
- Dr. Cooper, co-mentor, Michele and Howard J. Kessler Distinguished Chair in Surgery and Public Health; Kessler Director, Center for Surgery and Public Health; Director, Center for Geriatric Surgery; Chair of the Executive Advisory Council for Diversity, Equity, Inclusion and Community Health for Brigham and Women's Hospital, Associate Surgeon Division of Trauma, Burn and Surgical Critical Care, Brigham and Women's Hospital; Professor of Surgery, Harvard Medical School. She focuses on geriatric and palliative processes of care on postop outcomes of seriously ill older surgical pts. She has been well funded and published.

- Dr. Vranceanu (PhD in Clinical Health Psychology), post-doctoral fellow in Behavioral Medicine and Mind Body Research Fellowship; Associate Professor of Psychology in the Department Psychiatry at Harvard Medical School, Founding and directing the Center for Health Outcomes and Interdisciplinary Research (CHOIR) in the Department of Psychiatry at the MGH, and the Elizabeth and James E. Gambles Endowed Chair Co-Director of Massachusetts General Neurosciences (MGN; Co-Chair, International Consortium for Musculoskeletal Mental and Social Health; Chair, Finances and Funding, International Society of Neuro-palliative Care; Co-Director, Grants Consultations and Support, Massachusetts General Hospital; Faculty, ADRD Clinical Trials Intensive, Palliative Care Cooperative Group; Chair, Spirituality and Integrative Medicine Special Interest Group, Society of Behavioral Medicine; Co-Director, Palliative Care Clinical Trials Intensive Course. She has published over 230 peer reviewed articles. She is PI on more than 30 federal and foundation grants including seven R01s. She has expertise in qualitative methods (interview design, conduct, data coding and analyses), individual and dyadic biopsychosocial intervention development (using qualitative methods to design or adapt interventions), single-site and multi-site virtual or fully remote clinical trials. She has extensive mentoring expertise (~65). She holds a K24 award to mentor junior investigators in intervention development and clinical trials. She has been working with Dr. Cauley for over 1.5 years and serves as co-mentor on her Kornfeld grant. She runs the K club, which offers support to junior investigators preparation of grant application.

Weaknesses

- None noted.

5. Environment:

Strengths

- The institution has a strong research environment.

Weaknesses

- None noted.

Protections for Human Subjects:

Acceptable Risks and Adequate Protections.

Inclusion Plans:

- Sex/Gender: Distribution justified scientifically.
- Race/Ethnicity: Distribution justified scientifically.
- Inclusion/Exclusion Based on Age: Distribution justified scientifically.

Training in the Responsible Conduct of Research:

Acceptable.

Comments on Format:

- Format is included.

Comments on Subject Matter:

- Subject matter is included.

Comments on Faculty Participation:

- Faculty participation is included.

Comments on Duration:

- Duration is included.

Comments on Frequency:

- Frequency is included.

Resource Sharing Plans:

Unacceptable.

- Plans were not included.

Budget and Period of Support:
Recommend as Requested.

CRITIQUE 2:

Candidate: 1
Career Development Plan/Career Goals/Plan to Provide Mentoring: 3
Research Plan: 3
Mentor(s), Co-Mentor(s), Consultant(s), Collaborator(s): 2
Environment and Commitment to the Candidate: 1

Overall Impact:
This is a submission of an application for a Mentored Patient Oriented Research Career Development Grant (K23) from MGH to support Dr. Cauley for five years. The candidate is an Assistant Professor of Surgery, and a fellowship trained colorectal surgeon with expertise in diverticulitis management and surgical health outcomes. Dr. Cauley's research evaluates patient, clinician, and systems factors associated with health outcomes of patients undergoing surgery and with focus on health outcomes of older adults with serious illness. In this application, she is focused on development and rigorous testing of an intervention to improve integration of geriatric principles into surgical care of older adults undergoing fecal ostomy surgery. Seeks additional training in 3 major areas: 1) specialty training in clinical geriatrics related to ostomy needs; 2) the integration of qualitative and quantitative data in mixed methods research for intervention development and refinement; and 3) expertise in clinical trial methodology with particular focus on efficacy and effectiveness trial design for intervention evaluation. The candidate is outstanding with an impressive early trajectory, including leadership and productivity as a junior faculty. The primary mentor Dr. Ritchie is excellent, mentorship is outstanding, and the research environment and institutional support is also felt to be outstanding.

1. Candidate:
Strengths
- Dr. Cauley is an Assistant Professor of Surgery, Department of General and Gastrointestinal Surgery, MGH.
- Dr. Cauley completed medical school at Indiana School of Medicine, an MPH in clinical effectiveness at Harvard, and residency training in surgery at Harvard followed by a fellowship in colon (2018) and rectal surgery (2019).
- Dr. Cauley's research evaluates patient, clinician, and systems factors associated with health outcomes of patients undergoing surgery of the gastrointestinal tract and with focus on health outcomes of older adults with serious illness.
- KL2/CMERIT Harvard Catalyst Award Recipient and past Kornfeld Scholar.
- Spark grant PI that seeks to: Improving Surgical Recovery through High Quality Integration of mHealth Technology. The candidate was an investigator for a FOUNDATION grant entitled: Addressing Surgical Resource Needs in Health Care Systems' COVID-10 Response.
- 47 peer-reviewed publications.
- The candidate is first author on a qualitative study with Dr. Cooper.

Weaknesses
- One publication with Dr. Ritchie, the primary mentor.

2. Career Development Plan/Career Goals and Objectives:
Strengths
- Dr. Cauley describes her training goals as, 1) understand the unique needs of older adults with diverticulitis and how to address these needs with a tailored geriatric assessment and

management framework in the elective surgical setting, 2) strengthen methodologic skills in use of mixed methods for intervention development and refinement, and 3) acquire knowledge and experience in clinical trials focusing on pragmatic trials to rigorously evaluate the implementation of innovative surgical care interventions in the context of learning health systems.
- Dr. Cauley seeks additional training in: 1) specialty training in clinical geriatrics; 2) mixed methods research to develop and refine interventions; and 3) expertise in clinical trial methodology with particular focus on efficacy and effectiveness trial design for intervention evaluation.
- Table 1 in the CDP includes frequency of meetings. To understand the needs of older surgical patients, includes weekly MGH Perioperative Senior Health Clinic, monthly MGH Geriatrics Research Meetings, and quarterly American College of Surgeons (ACS) Geriatric Surgical Verification. Meetings include R01 grant writing course. Table 2 includes important detail on how career development activities fit with training aims by year.
- Includes clinical experiential training, clinical trials coursework, and weekly didactics from Dr. Ritchie (mentor) and early career intervention development and clinical trials group.
- Includes a table with timeline of training and professional development. Milestones included.
- Includes geriatrics tutorials with mentor and grand rounds.

Weaknesses
- None noted.

3. Research Plan
Strengths
- This application focuses on development and rigorous testing of an intervention to improve integration of geriatric principles into surgical care of older adults undergoing surgery.
- The candidate's aims are: SA1: to develop the first version of the GAM-oSurg intervention and methodology through 1:1 interviews with older adults 30-90 days after fecal ostomy surgery (up to N=30); SA2: to examine acceptability of GAM-oSurg via an open pilot (N=12 patients, N=4 surgical clinicians) with exit interviews and pre-post assessments. SA3: examine the feasibility of GAM-oSurg versus enhanced usual surgical care (N=48 patients per study arm) in a pilot RCT following predetermined benchmarks.
- Interview participants included diverse professions and occupations.
- Plans for an R01 efficacy trail included.
- Includes RA time for help with interviews.
- As strength is that it includes a detailed conceptual framework. The conceptual framework acknowledges caregiver burden as an outcome.

Weaknesses
- The conceptual framework is a strength as stated above, but it is hospital based and does not acknowledge the community setting where transitions happen (e.g., facility). It is unclear what the post-operative management encompasses in the framework as it is underdeveloped. Community should also label the third square that surrounds the post-operative management section of the framework.
- Preliminary data conducted by the team shows that one in six targeted patients is discharged to a facility and not at home, but interviews or key stakeholders do not include this or consider this important finding.
- Will include only patients in qualitative interviews and sample will include older adults but no mention of caregivers or family in the RP. States that that will be a focus of future studies - missed opportunity. An expected outcome is to publish a conceptual framework but without caregivers and families, the framework would be missing an important domain. One of the overall goals is to improve quality of life (QoL) and supporting autonomy and preferences but no caregivers are included.

- It is unclear how the surgeons will be recruited. States they will do national recruitment, so seems like one-on-one interviews would be better suited. Recruitment of surgeons, geriatricians, nurses, social workers, and physical therapists is not well detailed and underdeveloped. They are major stakeholders and end users.
- Unclear why non-English speaking patients will not be included. Even if the intervention will eventually be delivered in English, if scaled up it would be delivered using interpreters to non-English speaking patients. The first aims are qualitative, and the inclusion of linguistically diverse patients comprises the validity of qualitative data.
- Unclear how co-morbidities or existing medical conditions associated with surgery, or other social determinants will or will not be included, other than education.

4. Mentor(s), Co-Mentor(s), Consultant(s), Collaborator(s):
Strengths
- Primary mentor is Dr. Ritchie, Professor of Medicine and the Kenneth L. Minaker Chair in Geriatrics, and Director of Research for the Division of Palliative Care and Geriatric Medicine at MGH. Excellent mentoring track-record.
- Co-Mentor. Dr. Vranceanu, Associate Professor of Psychology and the Founding Director of the Center for Health Outcomes and Interdisciplinary Research in the Department of Psychiatry. She is an expert in biopsychosocial intervention development, refinement and clinical trials for patients, caregivers, and dyads including older adults.
- Ki-Do Eum, PhD is a Senior Statistical Analyst.
- Includes and advisory board.
- Candidate includes a letter from chair of surgery.

Weaknesses
- None noted.

5. Environment:
Strengths
- Outstanding research environment with resource and mentorship.
- 60% FTE protected research time because candidate is a surgeon.

Weaknesses
- None noted.

Study Timeline:
Strengths
- Detailed table included and detailed in this revision and resubmission.

Weaknesses
- None noted.

Protections for Human Subjects:
Acceptable Risks and Adequate Protections.
- Appropriate.

Data and Safety Monitoring Plan:
Acceptable.
- Appropriate.

Inclusion Plans:
- Sex/Gender: Distribution justified scientifically.
- Race/Ethnicity: Distribution justified scientifically.
- Inclusion/Exclusion Based on Age: Distribution justified scientifically.

Training in the Responsible Conduct of Research:
Acceptable.
Comments on Format:
- Appropriate, includes course and mentored learning.

Comments on Subject Matter:
- Appropriate.

Comments on Faculty Participation:
- Appropriate.

Comments on Duration:
- Appropriate

Comments on Frequency:
- Appropriate.

Resource Sharing Plans:
Acceptable.
- Now included in the revision.

Budget and Period of Support:
Budget Modifications Recommended (in amount/time).
- Candidate has budgeted $218 per year and $2592 for Years 1-4 for professional society membership fees.

CRITIQUE 3:

Candidate: 1
Career Development Plan/Career Goals/Plan to Provide Mentoring: 1
Research Plan: 6
Mentor(s), Co-Mentor(s), Consultant(s), Collaborator(s): 1
Environment and Commitment to the Candidate: 1

Overall Impact:
The K23 candidate Dr. Cauley proposes training and RP to become an independent investigator focused on health and patient-oriented outcomes in older geriatric surgery patients. Specifically, she will develop an intervention for surgery teams that will address the geriatric needs and outcomes (including QoL, independence, and caregiver burden) of fecal ostomy surgery patients. Dr. Cauley is a very strong candidate with a significant record of research, publications, and research awards. The mentoring team is superb with required expertise and exceptional mentoring records. Training is focused on identified research/knowledge gaps and should prepare the candidate for R01 applications and independence. Concerns regarding the RP, in particular with the SA3 pilot RCT, significantly dampened enthusiasm.

1. Candidate:
Strengths
- Assistant Professor of Surgery (2019).
- MPH (Clinical Effectiveness; 2015).
- Colorectal surgeon, expertise in fecal ostomy management and surgical health outcomes.
- MyNCBI lists 47 publications since 2012, 16 as first author.
- Research evaluating patient, clinician, and system factors associated with health outcomes of older surgical patients; has some experiences with qualitative research as well as development and evaluation of a surgeon-patient communication tool.

- Has received research awards and funding – Kornfeld Scholars Program (2022; National Palliative Care Research Network); KL2 from Harvard CTSA (2022) for training in mixed methods research; Spark grant.
- Has conducted research and published with mentors.

Weaknesses

- None noted.

2. Career Development Plan/Career Goals and Objectives:

Strengths

- Long-term goal: Independent investigator in geriatric surgery to improve biopsychosocial outcomes of older surgical patients.
- Training goals and plans address clinical geriatrics (related to ostomy needs); mixed methods data integration relative to development and refinement of interventions; clinical trial methods.
- Training involves a nice mix of relevant courses; regular clinical and research rounds; topic-based work with mentors including literature reviews and conduct of the RP; and attendance at scientific meetings.
- Professional training includes leadership training and grant writing courses.
- Will devote 60% effort to K23 training and research aims.
- Identifies papers and presentations with research aims.
- Has a planned R01 that will conduct an efficacy trial of the intervention on patient QoL.

Weaknesses

- None noted.

3. Research Plan:

Strengths

- SA1: Develop initial version of a Geriatric Assessment and Management framework for ostomy Surgery (GAM-oSurg). The GAM-oSurg intervention entails identification and management of geriatric conditions by the surgical team pre- and post-surgery, in and out of the hospital.
- Approximate n=30 semi-structured interviews with patients 30-90 days following surgery.
- Focus groups with key clinical stakeholders, including surgeons, geriatricians, surgical nurses, social workers (n=24).
- Will use purposive sampling for diversity (by age, minority, and socioeconomic status).
- Intervention prototype uses elements of geriatric assessments, GAM strategies in oncology and neurology, other assessments, and input from local experts. Multidimensional assessment domains (mind, mobility, medication, and what matters most).
- SA2: will pilot test the instrument to assess acceptability with exit interviews and pre/post assessments (n=12 patients, in 2 surgical teams (6 pts each)); refining the instrument. Assessments will include quality of life, GAM-oSurg and client satisfaction (pre- and 6-weeks post-surgery in patient); geriatric knowledge (pre/post, clinicians. 1:1 structured exit interviews regarding acceptability, clarity to be used for intervention and training refinement. There is a well-described training protocol for the surgical teams.
- SA3: conduct a pilot RCT to assess the feasibility of GAM-oSurg versus enhanced surgical care (n=48 per arm).
- Enhanced surgical care = completion of a multidomain checklist by clinician.
- Feasibility outcomes include recruitment, randomization, intervention fidelity, retention; threshold benchmarks are defined for each.
- Other outcome data will be collected, including patient QoL; conditions identified and managed; clinician geriatric knowledge; acceptability.
- Outcomes collected pre-surgery and six weeks post-surgery.

Weaknesses

- It is not clear why a two-armed RCT is required for the primary feasibility outcomes in SA3. What is the need for the EUC arm?

- SA3 analyses do not consider multiple patients within surgical teams and how this will influence feasibility outcome estimates. In fact, estimates of within-team ICC of the efficacy outcome (patient QoL) is not indicated.
- SA3 sample size is poorly described. Parameters used to assess power and the resulting power are not provided.
- As described the conduct of the SA3 pilot RCT provides significant opportunity for intervention contamination. However, the unit of randomization is not clear. On page 84 of the application, randomization is described at the patient level (D.4.2) as well as at the surgical team level (D.4.4). On page 170, it seems that the patient is the unit of randomization. There is a concern statement regarding efforts to "avoid contamination" that is simply not believable – asking the clinicians to keep interventions and training confidential and use of fidelity checklists will not ensure that contamination does not occur for the next patient treated by this clinician.
- Will clinicians repeat assessments (e.g., the Geriatric Knowledge survey) for all patients? If so, how will these data be analyzed, given there will be some cumulative effects of the intervention? Are there other assessments that need to be similarly considered?
- D.4.4 says participants will be blinded. It is not clear what participants are meant here (patients, clinicians). Blinding is furthermore not possible.
- Enhanced usual care arm is poorly developed and not well described.

4. Mentor(s), Co-Mentor(s), Consultant(s), Collaborator(s):
Strengths
- Dr. Ritchie (primary mentor), geriatrician and palliative care physician, expertise in mixed methods and clinical trials. Director of Research (Division of palliative care and geriatric medicine); Director of Mongan Institute for Aging and Serious Illness. Research focuses on health and quality of life of older persons with chronic illness and comorbidities. Dr. Ritchie has an extremely strong and successful mentoring record; she has received mentoring awards. Has worked with Dr. Cauley since 2020, assisting with successful Kornfeld scholar and KL2 applications.
- Dr. Cooper, surgeon-scientist, trauma surgeon and palliative care physician, expertise in qualitative research. Directs the Center for Surgery and Public Health and the Center for Geriatric Surgery. Research on postoperative outcomes in seriously ill older surgical patients, perioperative experience of patient and caregivers, long-term patient-oriented outcomes. Has conducted research and published extensively with Dr. Cauley.
- Dr. Vranceanu, clinical health psychologist, expertise in development, testing and implementation of biopsychosocial interventions for patients and caregivers. Also provides expertise in qualitative research, mixed methods, and clinical trials. Holds a K24 mentoring award and has received numerous recognition awards for her mentoring.
- Collaborator Dr. Eum (statistics, health services research, clinical trials).
- Scientific advisory board includes expertise in geriatric surgery outcomes research, qualitative and mixed methods; intervention development and evaluation; surgical team interventions.
- There are excellent track records of mentoring and research funding.

Weaknesses
- None noted.

5. Environment:
Strengths
- Research will occur at MGH, with extensive training and research resources. There is access to recruit surgical patients and their caregivers as well as surgeons.
- Dr. Cauley is a core faculty researcher in the Ariadne Laboratory, with relevant training and research resources that include expertise in survey methods, qualitative and quantitative study design and analysis, and implementation science.
- Will utilize resources of the Harvard CTSA including grant review and grant writing support.

- Courses identified in the Harvard TH Chan School of Public Health.
- Department of Surgery has provided 60% protected time; MGH as provided unrestricted consulting with statisticians and programmers; access to Ariadne Laboratory datasets and researchers.

Weaknesses

- None noted.

Protections for Human Subjects:
Acceptable Risks and Adequate Protections.

Data and Safety Monitoring Plan:
Acceptable.

- Will use an internal DSMB for pilot RCT.

Inclusion Plans:

- Sex/Gender: Distribution justified scientifically.
- Race/Ethnicity: Distribution justified scientifically.
- Inclusion/Exclusion Based on Age: Distribution justified scientifically.

Target enrollment tables do not include surgical teams.

Training in the Responsible Conduct of Research:
Acceptable.
Comments on Format:

- In person and online.

Comments on Subject Matter:

- Topics detailed.

Comments on Faculty Participation:

- Senior faculty involved in in-person training; RCR discussions will also occur with mentors.

Comments on Duration:

- 8 hours in person; 4 hours online.

Comments on Frequency:

Resource Sharing Plans:
Unacceptable.

- No resource sharing plan.

Budget and Period of Support:
Recommend as Requested.

THE FOLLOWING SECTIONS WERE PREPARED BY THE SCIENTIFIC REVIEW OFFICER TO SUMMARIZE THE OUTCOME OF DISCUSSIONS OF THE REVIEW COMMITTEE, OR REVIEWERS' WRITTEN CRITIQUES, ON THE FOLLOWING ISSUES:

PROTECTION OF HUMAN SUBJECTS: ACCEPTABLE. No concerns were reported by the scientific review group pertaining to the compliance with the NIH regulations for the protection of human subjects.

INCLUSION OF WOMEN PLAN: ACCEPTABLE. No concerns were reported by the scientific review group regarding the enrollment of women.

INCLUSION OF MINORITIES PLAN: ACCEPTABLE. No concerns were reported by the scientific review group in relation to minority groups' recruitment.

INCLUSION ACROSS THE LIFESPAN: ACCEPTABLE. No concerns were reported by the scientific review group regarding the inclusion throughout the lifespan; children exclusion is scientifically justified.

COMMITTEE BUDGET RECOMMENDATIONS: The budget was recommended as requested.

Footnotes for 1K23AG084876-01; PI Name: Cauley, Christy Elaine

NIH has modified its policy regarding the receipt of resubmissions (amended applications).See Guide Notice NOT-OD-18-197 at https://grants.nih.gov/grants/guide/notice-files/NOT-OD-18-197.html. The impact/priority score is calculated after discussion of an application by averaging the overall scores (1-9) given by all voting reviewers on the committee and multiplying by 10. The criterion scores are submitted prior to the meeting by the individual reviewers assigned to an application, and are not discussed specifically at the review meeting or calculated into the overall impact score. Some applications also receive a percentile ranking. For details on the review process, see http://grants.nih.gov/grants/peer_review_process.htm#scoring.

MEETING ROSTER

Career Development for Clinicians/Health Professionals Study Section
National Institute on Aging Initial Review Group
NATIONAL INSTITUTE ON AGING
Carrer development for clinicians and health professional awards

AGCD-3
06/12/2023 - 06/13/2023

Notice of NIH Policy to All Applicants: Meeting rosters are provided for information purposes only. Applicant investigators and institutional officials must not communicate directly with study section members about an application before or after the review. Failure to observe this policy will create a serious breach of integrity in the peer review process, and may lead to actions outlined in NOT-OD-22-044 at https://grants.nih.gov/grants/guide/notice-files/NOT-OD-22-044.html, including removal of the application from immediate review.

[redacted]

[redacted]

* Temporary Member. For grant applications, temporary members may participate in the entire meeting or may review only selected applications as needed.

Consultants are required to absent themselves from the room during the review of any application if their presence would constitute or appear to constitute a conflict of interest.
